# Biological functions and therapeutic applications of human mucosal-associated invariant T cells

**DOI:** 10.1186/s12929-025-01125-x

**Published:** 2025-03-01

**Authors:** Ying Fang, Yuning Chen, Siyue Niu, Zibai Lyu, Yanxin Tian, Xinyuan Shen, Yan-Ruide Li, Lili Yang

**Affiliations:** 1 Department of Microbiology, Immunology and Molecular Genetics, University of California, Los Angeles, Los Angeles, CA 90095 USA; 2 Department of Bioengineering, University of California, Los Angeles, Los Angeles, CA 90095 USA; 3 Molecular Biology Institute, University of California, Los Angeles, CA 90095 USA; 4 Eli and Edythe Broad Center of Regenerative Medicine and Stem Cell Research, University of California, Los Angeles, Los Angeles, CA 90095 USA; 5 Jonsson Comprehensive Cancer Center, David Geffen School of Medicine, University of California, Los Angeles, Los Angeles, CA 90095 USA; 6 Parker Institute for Cancer Immunotherapy, University of California, Los Angeles, Los Angeles, CA 90095 USA

**Keywords:** MAIT cells, MR1, Innate-like lymphocytes, Unconventional T cells, Tissue-specific adaptations, Immune surveillance, Cancer, CAR-T cell therapy, Allogeneic CAR-T cells, Microbiota, Metabolites

## Abstract

Mucosal-associated invariant T (MAIT) cells are a unique subset of innate-like T lymphocytes that bridge innate and adaptive immunity. Characterized by their semi-invariant T cell receptor (TCR) and abundant localization in mucosal tissues, MAIT cells recognize microbial metabolites, primarily derived from the riboflavin biosynthesis pathway, presented by the major histocompatibility complex (MHC)-related protein 1 (MR1). This interaction, along with co-stimulatory signals, triggers rapid immune responses, including cytokine secretion and cytotoxic activity, highlighting their importance in maintaining immune homeostasis and combating infections. This review provides an in-depth overview of MAIT cell biology, including development, activation pathways, and functional diversity, highlighting their protective roles in immunity, contributions to diseases like cancer and inflammatory bowel disease (IBD), and context-dependent dual functions in health and pathology. This review also highlights the emerging therapeutic potential of MAIT cells in immunotherapy. Their unique TCR specificity, abundance, and tissue-homing properties make them ideal candidates for engineering novel therapies, such as chimeric antigen receptor (CAR)-MAIT cells, targeting infections, cancers, and autoimmune diseases. Challenges like antigen escape, T cell exhaustion, and CAR design optimization must be addressed to enhance clinical efficacy. In summary, MAIT cells are integral to immune function, and their therapeutic potential presents exciting opportunities for the treatment of a wide range of diseases. Further research is essential to unlock the full potential of these versatile immune cells.

## Introduction

Mucosal-associated Invariant T (MAIT) cells are an underexplored specialized subset of T cells that play a crucial role in mucosal immune responses, serving as a bridge between innate and adaptive immunity. Their unique biology and function are defined by the expression of a semi-invariant T cell receptor (TCR), composed of an α-chain variable region (Vα) 7.2 (TRAV1-2) and an α-chain joining region (Jα) 33 (TRAJ33) paired with a limited set of beta chains (TRBV6, TRBV13, TRBV19, or TRBV20), enabling MAIT cells to recognize microbial metabolites from the riboflavin biosynthesis pathway presented by the monomorphic MHC class I-related molecule, MHC-related protein 1 (MR1) [1–4]. The semi-invariant TCR of MAIT cells and their restricting element, MR1, are highly conserved across mammals, underscoring their non-redundant roles tied to antigenic specificity, especially in mucosal and blood where they represent a substantial proportion of T cells [4, 5].

The ability of MAIT cells to recognize a broad range of microbes, coupled with their abundance and rapid, innate-like effector functions, suggests that they play a key role in human immunity. As mentioned, the primary antigens recognized by MAIT cells are metabolic adducts from the riboflavin biosynthesis pathway. These metabolites are produced by many pathogenic bacteria, as well as members of the intestinal microbiota. Interestingly, these microbial ligands can cross epithelial barriers and circulate throughout the body. This indicates that MAIT cells may play a role in real-time immune surveillance of microbiota dysbiosis, even across intact epithelial barriers [5]. Beyond their microbial recognition, MAIT cells exhibit functional plasticity, producing diverse cytokines and cytotoxic molecules, such as IL-17, IL-22, IFN-γ, and Granzyme B, which enable them to modulate local immune responses, promote tissue repair, and maintain epithelial integrity [6]. However, their roles extend beyond infectious diseases: emerging evidence highlights their involvement in non-communicable conditions such as cancer, autoimmune diseases, and chronic inflammatory disorders [7–9]. These diverse roles in protection and pathology underscore the versatility of MAIT cells in immune regulation and their potential as therapeutic targets.

In this review, we examine multiple aspects of MAIT cells, from their biology and development to their roles in diseases and therapies. We discuss the phenotypic and functional diversity of MAIT cells, their interactions with the microbiota, and their contributions to immune surveillance and tissue homeostasis. Additionally, we explore the emerging therapeutic potential of engineered MAIT cells, particularly in cancer immunotherapy, highlighting recent advances and identifying critical areas for future research.

### Biology and development of human MAIT cells

MAIT cells are unique lymphocytes whose development begins in the thymus shortly after birth, where positive selection is mediated by MR1-expressing double-positive cortical thymocytes [3]. Emerging evidence suggests that this process may also involve selection by self-antigens presented in the thymus, MR1 in the absence of ligand binding, or microbial-derived antigens from commensal flora [10].

The selection mechanism of MAIT cells critically depends on signaling via the MAIT TCR alongside engagement of signaling lymphocytic activation molecule-associated protein on cortical thymocytes. Following positive selection, MAIT cell precursors progress through distinct developmental stages, transitioning from CD24⁺CD44⁻ to CD24⁻CD44⁻ through downregulation of CD24 and upregulation of transcriptional regulators such as KLF2, CD62L, and S1PR1, mirroring the differentiation trajectory of conventional T cells. This stage marks the initial expression of the lineage-defining transcription factor promyelocytic leukemia zinc finger (PLZF) [3, 11, 12]. Terminal differentiation yields CD24⁻CD44⁺ cells that further diversify into functional subsets characterized by the expression of T-bet (MAIT1 subset) or RORγt (MAIT17 subset). These MAIT cell subsets exhibit distinct functional profiles and tissue tropism: MAIT1 cells preferentially localize to the spleen, lymph nodes, and liver, where they respond robustly to danger signals by expressing natural killer (NK) cell-associated receptors and producing cytotoxic molecules, including interferon-γ (IFN-γ). In contrast, MAIT17 cells are enriched in barrier tissues such as the lung, skin, and gut, where they predominantly secrete IL-17 and IL-22, mediators implicated in tissue repair and homeostasis [11, 13].

MAIT cell development also depends on a range of regulatory factors, including cytokine signaling and thymic selection. During positive selection, MR1 presents antigens, such as vitamin B derivatives, driving progression through three distinct developmental stages. Stage 1 MAIT cells are characterized by CD3⁺MR1-tet⁺CD27⁻CD161⁻IL-18R⁻ markers. In stage 2, CD27 expression increases, transitioning the cells to a CD3⁺MR1-tet⁺CD27⁺CD161⁻ phenotype. Finally, stage 3 cells acquire high IL-18R expression, exhibiting the signature CD3⁺MR1-tet⁺CD27⁺/⁻CD161⁺IL-18R⁺ profile. Importantly, the use of MR1 tetramers is critical for accurately detecting MAIT cells, as monoclonal antibodies may misidentify Vα7.2⁺ non-MAIT cells or exclude earlier-stage MAIT cells. Notably, MAIT cells continue to mature and develop distinct gene expression profiles after exiting the thymus, influenced by their tissue localization. For instance, MAIT cells in the liver, lungs, and gut exhibit distinct functional profiles tailored to their respective environments. This ongoing maturation supports their functional specialization in immune surveillance and barrier tissue maintenance.

In summary, advances in understanding MAIT cell biology have clarified their developmental trajectory and highlighted the distinct characteristics of each stage. This progress has also emphasized the need for precise detection methods to accurately distinguish MAIT cells from non-MAIT T cells, further advancing research into their roles in health and disease.

### Phenotypic and functional characterization of human MAIT cells

Beyond developmental markers, additional characterization markers are essential for identifying and isolating MAIT cells, enabling subset distinction, activation state tracking, and exploration of functional roles in health and disease (Table 1). MAIT cells are broadly classified into circulating and mucosal subsets, each defined by distinct steady-state and activation phenotypes, which facilitate their differentiation and characterization. In humans, circulating MAIT cells within the peripheral blood exhibit a homogeneous expression of IL-18Rα, CD161, and CD26, which facilitates their identification via cytometry [4, 14]. These cells display an effector memory phenotype (CD45RO^+^, CD27^+^, CCR7^−^, CD44^high^, CD62L^low^) and express an array of integrins and tissue-homing chemokine receptors (CCR5^high^, CCR6^high^, CXCR6^high^, and CCR9^int^), indicative of their capacity for migration toward sites of local inflammation [2, 4, 6, 14–21]. In contrast, mucosal MAIT cells are distinguished by the co-expression of tissue-resident markers CD69 and CD103. Although they share the effector memory phenotype observed in circulating MAIT cells, mucosal subsets exhibit higher levels of cutaneous lymphocyte-associated antigen [22, 23].

**Table 1:** Phenotypic and functional markers of human MAIT cells

Name	Marker category	Functional significance	Expression level	References
CCR5	Characterization surface antigen	Tissue-homing chemokine receptors	High expression on circulating MAIT cells	[2, 4, 6, 14–21]
CCR6	Characterization surface antigen	Tissue-homing chemokine receptors	High expression on circulating MAIT cells	[2, 4, 6, 14–21]
CCR9	Characterization surface antigen	Tissue-homing chemokine receptor	Intermediate expression on circulating MAIT cells	[2, 4, 6, 14–21]
CD103	Characterization surface antigen	Tissue-resident marker, particularly in mucosal tissues	Positive on mucosal MAIT cells	[22, 23]
CD107a	Activation and functional surface antigen	Degranulation marker	Upregulated upon activation of MAIT cells	[25]
CD161 (KLRB1)	Characterization surface antigen	Surrogate marker; activating receptor expressed on various immune cells	High on mature MAIT cells	[4, 14]
CD24	Characterization surface antigen	MAIT development stage marker	High on Stage 1 (immature) MAIT cells in the thymus; low on stage (mature) MAIT cells	[3, 11]
CD25	Activation and functional surface antigen	IL-2 receptor α-chain; facilitates high-affinity IL-2 binding	Upregulated upon activation of MAIT cells	[30, 31]
CD26	Characterization surface antigen	Co-stimulatory molecule that activates T cells	High on mature MAIT cells	[4, 14]
CD27	Characterization surface antigen	Memory T cell marker	Positive on mature MAIT cells	[2, 4, 6, 14–21]
CD44	Characterization surface antigen	MAIT development stage marker	Low on Stage 1 and Stage 2 MAIT cells in the thymus; high on Stage 3 MAIT cells	[3, 11]
CD45RO	Characterization surface antigen	Memory T cell marker	Positive on mature MAIT cells	[2, 4, 6, 14–21]
CD69	Characterization and functional surface antigen	Tissue-resident and activation marker	Upregulated upon activation of MAIT cells	[22, 23]
CLA	Characterization surface antigen	Skin-homing marker	Positive on MAIT cell in the skins	[22, 23]
CSF2	Activation and functional cytokine	TCR-dependent activation marker; stimulates the differentiation and activation of myeloid cells	Produced upon TCR-dependent activation of MAIT cells	[33]
CXCR6	Characterization surface antigen	Tissue-homing chemokine receptors	High expression on circulating MAIT cells	[2, 4, 6, 14–21]
Granzyme B	Activation and functional cytotoxic molecules	TCR-independent activation; induces apoptosis via activation of caspases and degradation of intracellular proteins	Produced upon TCR-independent activation of MAIT cells; higher in CD8^+^ MAIT cells	[24]
IFN-γ	Activation and functional cytokine	Pro-inflammatory cytokine produced upon activation	Upregulated upon activation of MAIT cells	[41]
IL-12	Activation and functional cytokine	Pro-inflammatory cytokine involved in MAIT cell activation	Upregulated upon activation of MAIT cells	[41]
IL-17A	Activation and functional cytokine	TCR-dependent activation marker; promotes inflammation particularly in bacterial and fungal infections	Produced upon TCR-dependent activation of MAIT cells	[32]
IL-18	Activation and functional cytokine	Pro-inflammatory cytokine involved in MAIT cell activation	Upregulated upon activation of MAIT cells	[41]
IL-18Rα	Characterization cytokine receptor	Pro-inflammatory cytokine receptor	Highest in MAIT cells compared with other T cell populations	[121, 122]
Perforin	Activation and functional cytotoxic molecules	TCR-independent activation; facilitates apoptosis in infected or malignant cells	Produced upon TCR-independent activation	[24]
PLZF	Characterization and functional transcription factor	TCR-independent activation; transcription factor critical for the development and effector function of innate-like lymphocytes	Produced upon TCR-independent activation of MAIT cells	[3, 11]
RORγt	Characterization transcription factor	Transcription factor associated with Tc17 phenotype; promotes terminal differentiation	Higher in DN MAIT cells than in CD8^+^ MAIT cells	[32, 33]
T-bet	Characterization transcription factor	Transcription factor associated with Tc1 phenotype; promotes terminal differentiation	Higher in CD8^+^ MAIT cells than in DN MAIT cells	[30]
TNF	Activation and functional cytokine	TCR-dependent activation marker; mediates inflammation and apoptosis in infected or malignant cells	Produced upon TCR-dependent activation of MAIT cells	[33]
Vα7.2	Characterization surface antigen	Variable region of the TCR alpha chain	Positive on mature MAIT cells; representing about 90% of the MAIT cell population	[123]

MAIT cells also exhibit population heterogeneity based on CD4/CD8 expression. Circulating MAIT cells predominantly consist of CD8^+^ (~ 70%) and double-negative (~ 15%) subsets, with a low frequency of CD4^+^ MAIT cells (~ 5%) [24]. In contrast, the double-negative subset is enriched within mucosal tissues, comprising ~ 40–50% of the total MAIT cell population [24]. Functionally, CD8^+^ MAIT cells demonstrate superior pro-inflammatory potential, characterized by higher expression of CD16 and NKG2D, elevated production of TNF-α and IFN-γ, and increased cytotoxic capacity, as evidenced by Granzyme B content and CD107A expression upon stimulation [25].

### Activation mechanisms and functional regulation of human MAIT cells

#### Functional markers

MAIT cells can be activated through both TCR-dependent and TCR-independent mechanisms. In the TCR-dependent pathway, microbial metabolites presented by MR1 molecules, in conjunction with co-stimulatory signals from cytokines or toll-like receptors (TLRs), initiate MAIT cell activation. This enables MAIT cells to enhance local immune responses, including promoting macrophage-mediated phagocytosis and recruiting immune cells to sites of inflammation [24]. Additionally, MR1-expressing cancer cells can serve as direct targets for MAIT cells, suggesting a potential role in tumor surveillance. In TCR-independent pathways, MAIT cells can also be activated by pro-inflammatory cytokines such as IL-12, IL-18, and IFN-γ. This implies their involvement in antiviral defense, as studies have demonstrated the activation of CD161^+^Vα7.2^+^ MAIT cells in human peripheral blood during clinical infections with viruses such as dengue, hepatitis C, and influenza A [26]. The ability of MAIT cells to respond to viral infections without TCR engagement suggests a broader role in host defense beyond bacterial recognition.

When activated, MAIT cells upregulate specific surface markers and cytokines indicative of their activation. Notably, markers such as CD69 and CD25 are commonly associated with MAIT cell activation. CD69, a membrane-bounded C-type lectin receptor, is an early activation marker expressed on T cells, including MAIT cells [27]. Increased surface expression of CD69 has been reported in inflammation in autoimmune diseases. In particular, patients with ulcerative colitis exhibit a higher frequency of CD69^+^ MAIT cells compared to healthy controls, underscoring its role as a marker of immune activation in diseases [28]. Additionally, in psoriasis, elevated CD69 expression on circulating MAIT cells in patient peripheral blood further supports its utility as a key indicator of their activation [29]. Another important activation marker is CD25, which is also known as the IL-2 receptor alpha chain. Experimental findings have demonstrated that CD25 is upregulated following viral stimulation, serving as a reliable indicator of immune activation [30, 31]. In addition, the degranulation marker CD107a is also upregulated. The activation of MAIT cells not only involves surface marker upregulation but is also characterized by the production of various cytokines and cytotoxic molecules, which differ depending on the pathway involved. In TCR-dependent activation, rapid immune response is characterized by increased expression of RORγt and the production of cytokines such as IL-17A, TNF, and CSF2, indicative of a Tc17-like phenotype [32, 33]. In contrast, TCR-independent activation primarily induces the release of IFN-γ, Perforin, and Granzyme B, regulated by the transcription factors PLZF and T-bet, which are linked to a Tc1-like phenotype [30].

Studying these MAIT cell functional markers offers therapeutic potential in infectious diseases, cancer, and autoimmune disorders. Modulating TCR-dependent/independent, or cytokine-driven activation pathways could enhance immune responses or control inflammation, making MAIT cells promising candidates for immunotherapy across a range of conditions.

#### Transcription regulation

Upon activation, MAIT cells exhibit a robust production of cytokines and activation markers. This dynamic response is tightly regulated by specific transcription factors, which trigger the downstream signaling pathways responsible for MAIT cell effector functions (Table 2). A key regulator of MAIT cell function is the transcription factor RORγt, which controls the production of IL-17A [34]. Beyond MAIT cells, RORγt is essential for the differentiation of other immune cells involved in type 17 immunity, such as Th17 cells, gamma delta T cells, and type 3 innate lymphoid cells, highlighting its broad role in regulating immune responses [35]. Although RORγt expression is nearly universal among MAIT cells, only a small fraction of these cells produces IL-17A ex vivo, suggesting either a pre-committed IL-17-producing subset or a broader potential for IL-17 production under the appropriate conditions [14, 36].

**Table 2:** Key transcription factors, functional significance, and associated signaling pathways of human MAIT cells

Transcription factors	Functional significance	Related down-signaling pathways	References
AP-1	Regulates immune activation, proliferation, differentiation, and apoptosis; activated by TCR engagement, cytokines, and stress signals	Limited information; further research needed	[27, 38]
BATF	Promotes Th17 differentiation; enhances IFN-γ production in cytokine-stimulated MAIT cells	IFN-γ signaling pathway	[23, 40]
EGR1	Controls NKT lineage differentiation in response to TCR signaling	Limited information; further research needed	[124]
Eomesodermin	Highly expressed in CD8^+^ MAIT cells; critical for cytotoxic activity; evenly distributed between CD8^+^ and DN MAIT cells in mucosal tissues	Regulates cytotoxic mediators such as Granzyme B and Perforin	[38]
Helios	Controls immune tolerance; regulates Tregs; suppresses inflammation; prevents excessive activation	Limited information; further research needed	[11, 98]
NF-κB	Central regulator of inflammation, cell survival, and proliferation	AHR signaling pathway	[38]
PLZF	Required for MAIT cell maturation (stage 2 to 3); drives CD44 expression; regulates CCR6 expression	TCR-independent activation pathway (via IL-12 and IL-18 signaling)	[34]
RORγt	Controls IL-17 production; promotes a Tc17 phenotype; essential for IL-23 receptor expression	IL-17 signaling pathway	[23, 24, 60]
RUNX3	Regulates PLZF expression; involved in T cell differentiation, especially CD8^+^ T cells	May regulate TCR-independent activation via PLZF	[34]
T-bet	Promotes a Tc1 phenotype; regulates the production of IFN-γ	IFN-γ signaling pathway; IL-12 signaling pathway; NF-κB signaling pathway	[11, 38, 125]

PLZF plays a pivotal role in shaping the innate-like features of MAIT cells. Expressed early during MAIT cell development, PLZF establishes an effector-memory phenotype in these cells. It acts as a master transcription factor, guiding MAIT cells toward their characteristic rapid-response behavior upon activation [37]. PLZF is consistently expressed across MAIT cell subsets, including CD8^+^, CD4^−^CD8^−^ double negative (DN), and CD4^+^ cells, underscoring its central role in the functional maturation and maintenance of MAIT cells within the immune system [37].

T-bet is another critical transcription factor that modulates MAIT cell function, particularly shaping their Th1-like responses. During TCR-mediated activation, T-bet drives the transcription of genes involved in cytotoxicity and pro-inflammatory signaling, primarily promoting the production of IFN-γ, thereby strengthening MAIT cells’ ability to generate a robust immune defense. T-bet expression is notably higher in CD8^+^ MAIT cells compared to DN MAIT cells, highlighting its role in cytotoxic responses [38].

Eomesodermin, closely related to T-bet, is highly expressed in CD8^+^ MAIT cells and is pivotal for their cytotoxic function. It regulates the expression of cytotoxic molecules such as Granzyme B and Perforin, essential for MAIT cells’ ability to eliminate infected or malignant cells [39]. Upon activation, eomesodermin ensures the transcription of these effector molecules, thus linking TCR engagement to the cytotoxic arm of MAIT cell function. Unlike in peripheral blood, eomesodermin expression in mucosal MAIT cells, such as those in the endometrium, is more evenly distributed between CD8^+^ and DN subsets, suggesting a tissue-specific regulation of this transcription factor [38].

The transcriptional landscape of MAIT cells is not only dictated by their activation state but also by their tissue localization. Comparative analyses of MAIT cells in blood and tissue reveal distinct transcriptional profiles, particularly in organs such as the liver. Liver-resident MAIT cells demonstrate enhanced activity of the transcription factors AP-1 and NF-κB, both of which are instrumental in regulating inflammation and cytokine production [38]. AP-1, which includes proteins like FOS and JUN, and NF-κB are significantly upregulated in liver MAIT cells compared to their counterparts in the bloodstream. This elevation correlates with increased expression of genes associated with T cell activation, suggesting that liver-resident MAIT cells are primed for rapid immune responses. Additionally, the TCR-induced transcription factor EGR1 and RUNX3, which regulates tissue-resident memory cell differentiation, are more active in liver MAIT cells, further emphasizing the tissue-specific adaptations of these cells [40].

The transcriptional regulation of MAIT cell activation is a complex, multifaceted process governed by a combination of factors such as RORγt, PLZF, T-bet, and eomesodermin. These transcription factors not only define the cytokine and cytotoxic responses of MAIT cells but also facilitate their adaptation to specific tissue environments. Understanding how these transcriptional programs cooperate in different contexts provides valuable insights into the diverse roles of MAIT cells in immune surveillance, inflammation, and tissue-specific immunity.

### Tissue localization and immune surveillance of human MAIT cells

The distinct localization of MAIT cells across barrier and mucosal tissues is crucial to their function in immune surveillance. These cells are particularly enriched in mucosal sites, such as the gut, lungs, and liver, where they account for 20–40% of T cells compared to their smaller presence (1–4%) in peripheral blood [11, 41]. Their high abundance at these sites underscores their specialization in detecting microbial threats and preserving tissue integrity.

Studies suggest that the tissue-specific distribution of MAIT cells is guided by the chemokine receptor CCR6, which binds to its ligand CCL20 expressed in mucosal tissues and the liver [42]. This receptor-ligand interaction ensures their recruitment to key sites of immune surveillance, where their functionality is tailored to the local environment. In the liver, MAIT cells exhibit a distinct pattern of distribution, being dispersed throughout the sinusoidal environment and portal fields, unlike other T cells, which are more localized to specific regions [42]. Liver-resident MAIT cells express activation markers, including CD69, HLA-DR, and CD38, which prepare them for rapid responses to microbial and inflammatory stimuli [43–45]. Functionally, they secrete IFN-γ and IL-17 in response to IL-12 and IL-18 stimulation during TLR8 activation, contributing to antiviral immunity and the regulation of hepatic inflammation and fibrosis [43, 45, 46]. As the predominant IL-17-producing T cell population in the liver, MAIT cells drive hepatic inflammation and fibrosis by activating Kupffer cells and biliary epithelial cells to release proinflammatory cytokines and chemokines [43, 46]. These functions underscore their critical role in liver immunosurveillance and regulation of immune responses to pathogens and tissue injury.

Beyond microbial defense, MAIT cells contribute to maintaining mucosal tissue integrity. In the gut, they produce IL-22 and IL-17, which are vital for epithelial repair and barrier maintenance [6]. IL-22 promotes the survival and proliferation of epithelial cells and stimulates mucus production by goblet cells, thereby reinforcing the physical barrier against pathogens [47, 48]. Meanwhile, IL-17 regulates tight junction proteins, such as occludin, to minimize barrier permeability during epithelial damage [49]. These cytokines collectively strengthen the mucosal barrier and limit excessive inflammation by suppressing the activation of conventional proinflammatory T cells, such as Th1 and Th17 subsets [50]. Similar functions have been observed in other mucosal tissues, including the genital tract, where MAIT cells play a role in epithelial repair and maintenance [24].

**Fig. 1:**
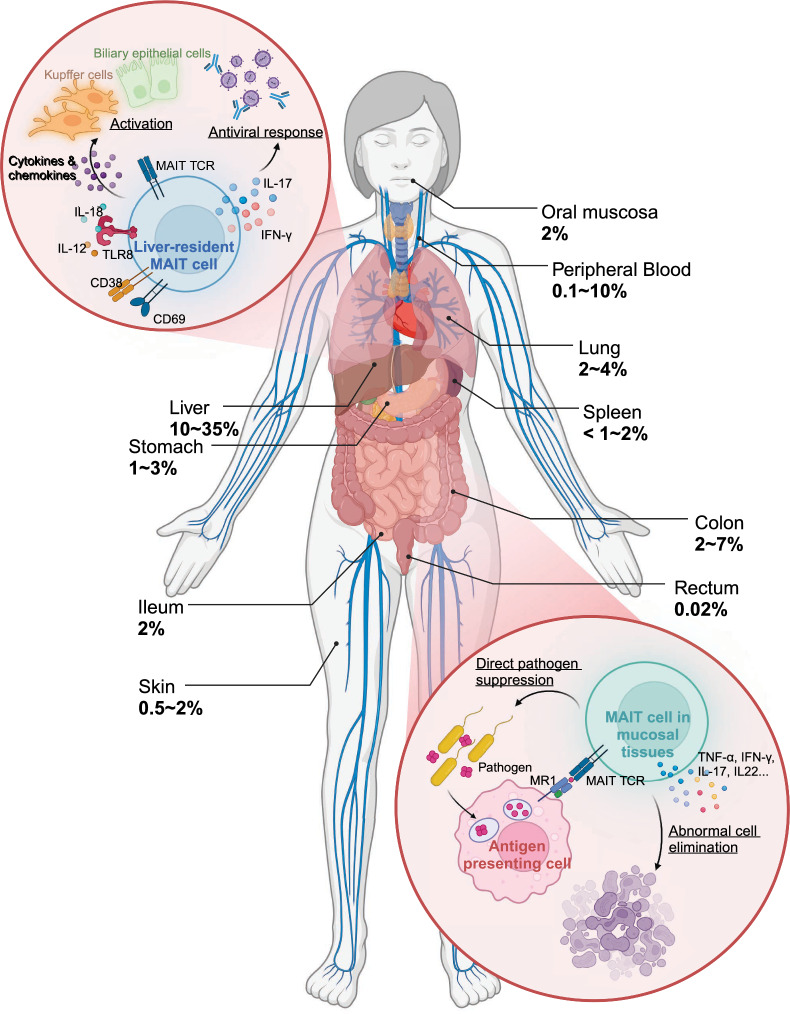
Tissue distribution and biological function of human MAIT cells. This figure illustrates the distribution of MAIT cells across various tissues in the human body. It shows the percentage of MAIT cells in each tissue, highlighting their localization and unique functional characteristics within the tissue microenvironment

Collectively, MAIT cells are uniquely positioned in barrier and mucosal tissues, where they act as key mediators of immune surveillance and response (Fig. 1). Their ability to produce a diverse array of cytokines and cytotoxic mediators enables them to combat microbial threats, maintain epithelial integrity, and regulate local immune activity. However, there are still knowledge gaps regarding the specific mechanisms driving MAIT cell localization in tissues. One promising approach to address this is studying the role of tissue-specific cytokines in polarizing MAIT cells toward pro-inflammatory or regulatory responses. For instance, exposing MAIT cells from various tissues to different cytokine combinations (e.g., IL-12/IL-18, IL-15/IL-18, IL-10, TGF-β) can reveal how the balance of pro-inflammatory and regulatory cytokines influences their function and role in inflammation [51]. Additionally, exploring the dysregulation of MAIT cell activation and tissue homing in autoimmune disease pathogenesis offers another critical avenue. In multiple sclerosis, for example, MAIT cells infiltrate the brain, producing pro-inflammatory cytokines that may contribute to neuroinflammation [52]. In IBD, although MAIT cell frequency is reduced in peripheral blood, they accumulate in inflamed mucosa, suggesting a role in intestinal inflammation and tissue repair [53]. However, MAIT cells may also contribute to the pathogenesis of IBD. Upon activation, MAIT cells upregulate NKG2D, an activation receptor for NK cells, which further induces the expression of proinflammatory cytokines [8]. Clinical trials have shown that treatment with anti-NKG2D mAb can induce clinical remission in some patients with CD, highlighting the role of NKG2D in CD pathogenesis [54]. Additionally, in an oxazolone-induced mouse model of colitis, inhibiting MAIT cell activation through MR1 knockout or administration of the MR1 antagonist isobutyl 6-formylpterin has been shown to mitigate the severity of UC [55]. Studies focusing on the mechanisms of MAIT cell recruitment and their production of regulatory cytokines like IL-10 and IL-22 could provide valuable insights into their protective roles in autoimmune diseases.

### Interactions between human MAIT cells and the microbiota

The immune system and the microbiota maintain a close, mutually dependent relationship. As a crucial part of the immune system, MAIT cells mainly respond to bacteria through TCR recognition of metabolites generated in the vitamin B2 (riboflavin) biosynthetic pathway [5]. Studies have demonstrated that disrupting or inhibiting riboflavin metabolism in various bacteria suppresses MAIT cell responses, confirming that this pathway produces antigens targeted by MAIT cells [56–59]. This connection demonstrates the selective nature of MAIT cell responses and their reliance on specific bacterial metabolic pathways.

Early-life exposure to the microbiota is crucial in shaping MAIT cell development and function. Research in germ-free mice has demonstrated that MAIT cells are absent or underdeveloped without microbial exposure [60]. Additionally, the timing of microbial colonization plays a critical role in MAIT cell maturation. For instance, recolonizing neonatal germ-free mice with early-life intestinal commensal bacteria before the third week of life promotes MAIT cell development, whereas colonization beyond this critical window fails to trigger the same result [60, 61]. This time-sensitive interaction highlights a developmental window during which exposure to specific microbial signals is essential for effective MAIT cell function. Although the microbiota composition differs between humans and mice, the principles governing MAIT cell development appear conserved [62]. Both species require exposure to microbial-derived riboflavin metabolites for MAIT cell maturation. Variability in early-life microbial exposures, influenced by factors like delivery mode and environment, likely contributes to the diversity in MAIT cell abundance observed in humans. Insights from germ-free mouse models highlight the translational potential of targeting riboflavin metabolites to modulate MAIT cell function in infections and inflammatory conditions.

Beyond development, MAIT cells are essential for maintaining mucosal homeostasis and ensuring protection against infections. They are strategically positioned at barrier sites, such as mucosal surfaces, where they provide localized immune surveillance and promote tissue repair following microbial disturbances [61]. For example, MAIT cells promote tissue repair in response to microbial disturbances. Through MR1-mediated presentation of riboflavin, MAIT cells recognize skin commensals, facilitating cutaneous wound healing and contributing to homeostasis. In the presence of commensal organisms, these cells activate tissue-repair pathways, supporting the maintenance of epithelial integrity and barrier function [60]. Their interaction with the microbiota ensures mucosal health by sustaining a balanced immune response and preventing pathogen colonization.

### Human MAIT cells in health and disease

MAIT cells play a dual role in health and disease, contributing to both homeostasis and pathology. In a healthy state, MAIT cells maintain tissue integrity and immune balance, but their dysfunction is linked to dysbiosis—disruptions in microbial composition and diversity—which is implicated in diseases such as cancer, inflammatory bowel disease, obesity, and metabolic disorders [63–66].

#### Gut diseases

The gut microbiota and MAIT cells interact in a bidirectional relationship that shapes immune responses in the intestinal mucosa. For instance, *Bacteroidetes*, abundant in the gut, strongly stimulate MAIT cells and influence their phenotype [67]. MAIT cells, in turn, protect the gut by recognizing riboflavin-derived metabolites presented by the MR1 molecule, allowing rapid responses to microbial challenges. These responses include cytokine release (e.g., IL-17 and IL-22), which maintains epithelial barrier function and promotes tissue repair [68]. However, there is also evidence showing the conflicting role of MAIT cells in gut diseases. In Crohn’s disease, MAIT cells accumulate in the ileal mucosa and exhibit a pro-inflammatory Th17 phenotype, secreting high levels of IL-17 and reduced IFN-γ upon stimulation [28]. In ulcerative colitis, MAIT cells are enriched in the colonic mucosa and display increased CD69 expression alongside elevated production of IL-17 and IL-22, correlating with disease activity [28]. These findings suggest that while MAIT cells contribute to gut protection, their dysregulation may drive chronic inflammation. The mechanisms by which MAIT cells balance protective immunity and inflammation in gut diseases remain poorly understood.

#### Autoimmune disorders

Beyond the gut, MAIT cell dysfunction is implicated in systemic autoimmune diseases, where altered microbial antigens and pro-inflammatory cytokines drive abnormal activation. This activation may lead to target cell killing and recruitment of other immune cells, disrupting immune tolerance and exacerbating inflammation [8]. In multiple sclerosis, peripheral MAIT cell levels exhibit dynamic fluctuations, decreasing during remission, further declining during relapses, reaching lower levels in active disease compared to stable cases, and increasing during clinical recovery [52]. This reduction may result from IL-18-driven activation and infiltration of CD8^+^ MAIT cells into the central nervous system [69]. Supporting this hypothesis, multiple sclerosis lesions exhibit massively expanded and persistent T cell populations that express canonical or atypical MAIT cell-related α chains compared to healthy tissue samples [70]. Similar fluctuations are observed in systemic lupus erythematosus, rheumatoid arthritis, and Sjögren’s syndrome, where altered MAIT cell activation and presence contribute to disease progression. These patterns highlight the central role of MAIT cells in autoimmune pathology and their potential as therapeutic targets [71–77].

#### Cancer

MAIT cells exhibit dual roles in cancer, acting as both protective and pathological agents depending on the cancer type and microenvironment. In mucosal-associated cancers, such as colorectal cancer (CRC), MAIT cells are enriched in malignant tissues compared to adjacent healthy tissue [7, 78, 79]. However, they are found at lower frequencies in CRC liver metastases [80], suggesting distinct patterns of infiltration between primary and metastatic sites. In blood cancers like multiple myeloma, patients exhibit reduced circulating MAIT cells and impaired IFN-γ production [81, 82]. This phenomenon may result from generalized lymphocytopenia commonly associated with blood cancers, the replacement of healthy cells by malignant ones, the depletion of MAIT cell precursors, or, in rare cases, the malignant transformation of MAIT cells themselves [7, 81–84].

Given the complex role of MAIT cells in cancer, they may also influence the efficacy of cancer therapies, such as chemotherapy, immune checkpoint inhibitors, and chimeric antigen receptor (CAR)-engineered cell therapy. The variability in MAIT cell responses and characteristics across different cancer types underscores the complexity of their role in cancer immunity. For example, in hepatocellular carcinoma, MAIT cells are found at lower frequencies in tumors compared to healthy tissues, and this reduction is associated with poor prognosis, suggesting a potential anti-tumor role for MAIT cells [85]. However, in the same context, MAIT cells exhibit increased expression of inhibitory receptors, including PD-1, CTLA-4, and TIM-3, which drive T cell exhaustion and impair their anti-tumor function [7, 86]. Additionally, these cells can produce elevated levels of pro-tumorigenic IL-8, facilitating the recruitment of immunosuppressive myeloid-derived suppressor cells into the tumor microenvironment [87, 88]. In CRC, an increased presence of MAIT cells within tumor sites correlates with poor prognosis, suggesting a tumor-promoting role for these cells [78]. This heterogeneity suggests that MAIT cells could either support or hinder therapeutic outcomes, depending on the specific cancer context and therapeutic approach. Further research is needed to clarify how MAIT cells can be modulated to enhance therapeutic efficacy.

### Human MAIT cells for therapeutic applications

#### Cancer

The development of adoptive T cell therapy in the past two decades has plenished the engineering strategies to harness immune cells to mediate antitumor and antiviral responses. MAIT cells possess unique potential for cancer treatment with their semi-invariant TCR and mucosal tissue-homing properties. While studies in mouse models have shown that 5-OP-RU stimulation enables MAIT cells to suppress tumor metastasis through an NK cell-associated, IFN-γ, and TNF-mediated mechanism, the role of MAIT cells in human cancer immunity is more complicated [89]. Their activation depends heavily on MR1-expressing antigen-presenting cells, which may limit their direct tumoricidal capabilities. However, in cancer with distinct traits of microbiota like CRC, bacterial antigens are extensively presented and MAIT cells could be activated subsequently. For instance, a CRC-related strain, *Fusobacteria nucleatum*, is identified with the capability to activate MAIT cells in a TCR-dependent manner [90].

**Fig. 2:**
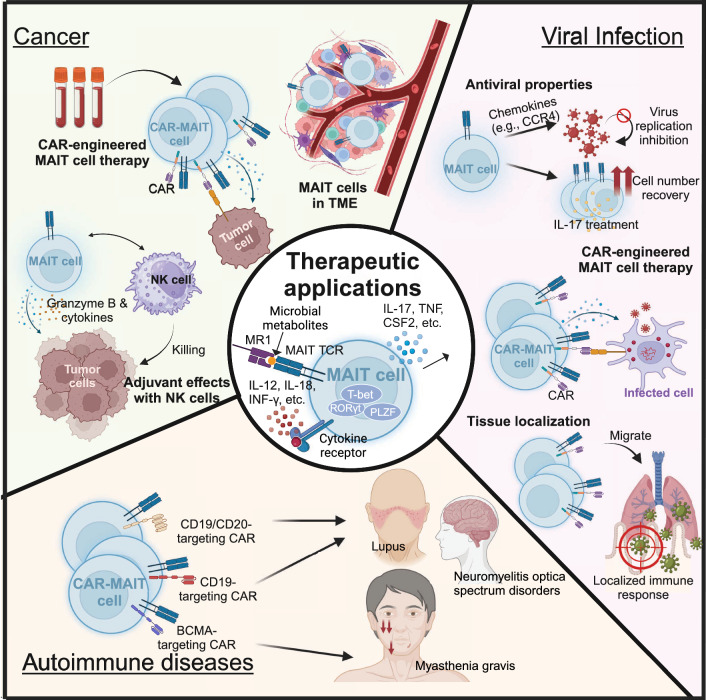
Therapeutic applications of human MAIT cells. This figure highlights potential therapeutic applications of MAIT cells in three key areas: cancer, viral infections, and autoimmune diseases. Each area is examined in detail, emphasizing the unique characteristics of MAIT cells that make them suitable for therapeutic use and their potential for clinical innovation. TME, tumor microenvironment

To date, current research still indicates that TCR-mediated MAIT cell activation is strictly dependent on MR1-vitamin B-related antigen (VitBAg). MR1 is considered monomorphic and exhibits limited genetic variation between individuals, which is unlikely to be a major target for allogeneic recognition by MAIT cells, resulting in a mitigated allogeneic response for cell therapy. However, the MR1-VitBAg and antigen presenting cell-dependent manner is also limiting the tumoricidal capability of MAIT cells since they can only recognize MR1-restricted tumoral antigens. Engineering MAIT cells with CAR could enhance their direct tumoricidal capability. The expanding repertoire of CAR designs and combinational therapies has addressed major challenges in CAR-T efficacy, including tumor microenvironment infiltration and delivery strategies, making them highly compatible with MAIT cells [91]. Meanwhile, the unique properties of MAIT cells highlight the advantages of engineering CAR-MAIT cells for cancer immunotherapy (Fig. 2). First, MAIT cells are prevalent in peripheral blood, where they exhibit mostly tumoricidal phenotype. Second, MAIT cells have highly restricted TCR dependent on MR1-VitBag, the activation of CAR-MAIT cells could be regulated by administration of agonists. MR1 expression in host cells is considered monomorphic, which could minimize allogeneic rejection of CAR-MAIT cells. Third, MAIT cells are potential adjuvant to initiate NK cell-mediated anti-tumor immunity. The crosstalk between CAR-MAIT cells and NK cells could result in multiple tumoricidal mechanisms.

In one study, introducing human EGF receptors and CD19 CARs to MAIT cells resulted in cytotoxic activity comparable to CAR-CD8 T cells in vitro, but with reduced production of IFN-γ and GM- CSF, cytokines associated with CAR-T cell function and cytokine release syndrome (CRS) [92]. This raises the question of whether CAR-MAIT cells could mitigate the side effects of CRS while preserving their antitumor efficacy. Meanwhile, advances in induced pluripotent stem cell (iPSC)-derived MAIT cells paved another route for CAR-MAIT manufacture [93]. Adoptive transfer of iPSC-derived murine MAIT cells prior to tumor inoculation significantly protected mice from metastasis formation and resulted in prolonged survival. Notably, the cytotoxicity of MAIT cells against tumor cell lines was observed in the absence of 5-OP-RU. In vivo depletion of NK cells could nullify the anti-tumor effects of MAIT cells, emphasizing the critical role of NK cells in this therapy.

MAIT cells have been found in the tumor microenvironment of a variety of solid tumors, including colorectal cancer, hepatocellular carcinoma, lung cancer, gastric cancer, kidney cancer, and brain cancer, with elevated tumor-infiltrating MAIT cells observed in colorectal cancer and hepatocellular carcinoma [2, 86, 90, 94]. Despite this, most of these tumor-infiltrating MAIT cells display an activated and exhausted phenotype, characterized by increased expression of activation markers (HLA-DR, CD38) and immune checkpoint molecules (PD-1, CTLA-4, TIM-3). Similarly, in hematological malignancies such as multiple myeloma, MAIT cells in peripheral blood show increased PD-1 expression. Importantly, in vitro PD-1 blockade restored MAIT cell function, suggesting that MAIT cell-based therapies could potentially benefit from strategies involving immune checkpoint blockade [81].

#### Viral infections and other diseases

MAIT cells are key players in antiviral immunity, but their responses vary depending on the infection type and stage. In chronic viral infections, including HIV, HBV, HCV, and HDV, MAIT cell frequencies are consistently reduced, with this decline persisting even after antiviral therapy, suggesting irreversible dysfunction [95–98]. Despite these challenges, MAIT cells demonstrate antiviral activity, as shown in vitro where they restrict HIV infection via upregulation of antiviral chemokines like CCL4 [95]. In acute viral infections, MAIT cell responses differ. For example, during acute HIV infection, MAIT cell counts initially increase but decline as the infection becomes chronic [95]. Also, MAIT cells are activated in acute viral infections such as Dengue virus (DENV) and Zika virus (ZIKV) [26, 99]. DENV infection in humans has been associated with heightened MAIT cell activation, as indicated by increased CD38 and Granzyme B expression, while only a slight reduction in MAIT cell frequency was observed in the blood [26, 99, 100]. In in vitro ZIKV infection, MAIT cells are activated and express IFNγ in an IL-18-dependent manner [99, 100].

Moreover, although human lung contains relatively fewer MAIT cells compared to liver, these cells can exhibit both protective and pathogenic functions in viral infections affecting the respiratory tract [101]. In humans, MAIT cell activation in response to influenza virus-infected lung epithelial cells in vitro is mediated by IL-18, which induces the expression of IFN-γ and Granzyme B, and higher level of MAIT cells has been correlated with better clinical outcomes [30, 99, 102]. Similarly, SARS-CoV-2 infection leads to both MAIT cell activation and depletion in peripheral blood. Notably, IL-7 treatment has shown potential in reversing MAIT cell dysfunction in SARS-CoV-2 [103], offering a glimpse into how therapeutic interventions might enhance MAIT cell function. However, in patients with severe infection of SARS-CoV-2, elevated MAIT cell activation levels are also associated with and serve as a predictor of mortality [99, 104–106]. Flament et al. reported that in severe COVID-19, the MAIT cell phenotype undergoes a progressive shift from a type I IFN immune profile to an IL-18 immune environment, mediated by a transcriptional switch in monocytes and macrophages, which suggests that IL-18 can drive both protective and pathogenic MAIT cell responses, depending on the disease context [105]. These findings underscore both the antiviral potential of MAIT cells and the challenges associated with their dysregulation in viral infections.

Building on this understanding, advances in immune engineering offer opportunities to enhance MAIT cell activity and direct their functions for therapeutic applications [13]. The success of CAR-T cell therapy in treating cancers and viral infections provides a robust framework for exploring engineered MAIT cells. CAR-T cells are engineered to express synthetic receptors that enable precise targeting of antigens without MHC dependence. By applying similar principles, MAIT cells could be engineered to express CARs that specifically recognize viral antigens, amplifying their natural antiviral activity and enabling them to target infected cells more effectively [13]. Preliminary studies have demonstrated the feasibility of generating CAR-engineered MAIT cell, highlighting their potential as a novel approach for combating viral infections [107]. MAIT cells’ unique tissue-homing properties and responsiveness to microbial antigens offer distinct advantages for engineering. For example, CAR-MAIT cells could be designed to localize in mucosal tissues where viral replication often occurs, providing targeted immune responses with reduced systemic toxicity. Additionally, their potential adaptability to chronic infections, such as HIV, or acute infections, like SARS-CoV-2, highlights their versatility.

Beyond antiviral applications, engineered MAIT cells offer exciting possibilities for autoimmune diseases and cancers with microbial component involvement [90]. Their unique ability to respond to microbial-derived antigens could be harnessed to modulate immune responses in inflamed tissues or target tumors with microbial components. The success of CAR-T cell therapy in autoimmune diseases highlights the potential of CAR-based approaches in modulating immune activity. For example, BCMA-targeting CAR-T cells are being investigated for myasthenia gravis, while anti-CD19 and dual anti-CD19/anti-CD20 CAR-Ts are being tested for lupus and neuromyelitis optica spectrum disorders [108–110]. Building on these advancements, the integration of MAIT cell biology with immune engineering could unlock innovative therapeutic strategies. However, further research is needed to optimize CAR designs for MAIT cells, enhance their isolation and expansion, and address challenges such as T cell exhaustion and antigen escape. Leveraging the innate-like properties of MAIT cells presents a potential strategy for enhancing tumor targeting. As a subset of innate-like T cells, MAIT cells express a semi-invariant TCR that recognizes MR1, which has been reported to be upregulated in certain malignancies, including myeloid leukemias and some epithelial cancers. Additionally, MAIT cells express NK receptors (e.g., NKG2D), enabling them to recognize stress-induced NK ligands such as MICA/B and ULBP family members on tumor cells. Notably, tumor cells may upregulate these ligands following treatment with hypomethylating agents or DNA-damaging chemotherapy, enhancing their susceptibility to MAIT cell-mediated cytotoxicity. This suggests a potential “triple-targeting” mechanism, integrating CAR, MAIT TCR, and NK receptor-mediated recognition, similar to strategies demonstrated in iNKT cells [111]. In addressing T cell exhaustion, the addition of cytokines such as IL-15 to the CAR construct could provide a promising solution. IL-15 is known to promote the survival, proliferation, and memory formation of T cells, while also enhancing the functionality of innate-like T cells, including MAIT cells [111]. This could mitigate the effects of exhaustion by maintaining the persistence and activity of CAR-MAIT cells in vivo, ultimately improving their therapeutic efficacy. Further investigations are necessary to optimize CAR designs, determine the ideal cytokine combinations, and evaluate the long-term safety and efficacy of CAR-MAIT cells in preclinical and clinical settings.

However, MAIT cells play a complex role in autoimmune diseases, contributing to both inflammation and immune regulation. They produce pro-inflammatory cytokines, including IL-17, TNF-α, and IFN-γ, which drive tissue damage in conditions such as rheumatoid arthritis, ankylosing spondylitis, psoriatic arthritis, and IBD, where IL-17 is a key pathogenic factor [112–114]. MAIT cells also express homing receptors that facilitate their accumulation in inflamed tissues, such as the synovial fluid in arthritis and the intestinal mucosa in IBD, further exacerbating inflammation [115, 116]. Additionally, MAIT cells can disrupt immune tolerance by responding to non-microbial antigens, leading to abnormal immune activation. In diseases like systemic lupus erythematosus and dermatomyositis, MAIT cells undergo activation-induced cell death, suggesting a potential role in disease progression [72]. These complexities pose challenges for the use of MAIT cells as therapeutic agents in autoimmune diseases. While their regulatory and tissue-repair functions make them attractive candidates, their pro-inflammatory properties raise concerns about exacerbating disease pathology. Thus, a deeper understanding of MAIT cell function in different autoimmune contexts is essential before considering their therapeutic application.

### Future directions

Despite significant advancements in understanding MAIT cells, many critical aspects remain unexplored, offering opportunities for further research and therapeutic innovation. A key area of interest is uncovering the tissue-specific roles of MAIT cells. These cells are found across mucosal tissues, the liver, and blood, but how their functions adapt to different microenvironments remains unclear [24, 117]. Investigating these variations could provide important insights into how tissue-specific factors influence their roles in immune surveillance and regulation, which could inform disease treatment strategies.

Another challenge lies in understanding the balance between the protective and pathogenic roles of MAIT cells. While they contribute to tissue repair and barrier integrity, their pro-inflammatory and cytotoxic activities can lead to immune-mediated damage [118]. Deciphering the molecular mechanisms that regulate this balance, including the influence of the microbiota, is crucial. Targeting microbiota-driven pathways could offer innovative strategies to modulate MAIT cell activity, particularly in conditions involving excessive inflammation or fibrosis.

Furthermore, MAIT cells also play complex roles in diseases such as cancer and viral infections. Their dual functions—cytotoxicity via IFN-γ production and tissue repair through IL-22—can either suppress or promote disease, depending on the context [119]. Signals from the tumor microenvironment or tumor-associated bacteria could influence these outcomes, offering potential therapeutic insights. In viral infections like COVID-19, MAIT cells display dynamic redistribution and cytokine responses, suggesting roles in both inflammation and recovery [119]. Understanding these mechanisms, especially in non-bacterial contexts, is vital for unlocking the potential of MAIT cells in these diseases.

Finally, as interest in immunotherapy continues to grow, particularly in the fields of cancer and infectious diseases, the therapeutic potential of MAIT cells is gaining significant attention. One promising approach is optimizing MAIT cell-targeted therapies, including vaccines designed to selectively activate these cells without triggering excessive inflammation. Such therapies could enhance mucosal immunity, providing a more focused and effective immune response. Additionally, the development of CAR-MAIT cells—engineered MAIT cells expressing chimeric antigen receptors—offers exciting possibilities for targeting specific antigens on cancer cells or infected tissues [120]. This approach could combine the tissue-repairing properties of MAIT cells with the precision of CAR-based therapies, expanding their potential in treating a range of diseases. By utilizing the unique features of MAIT cells, such as their rapid response to infection and tissue damage, CAR-MAIT therapy could provide a powerful tool for both cancer immunotherapy and infectious disease management. Moving forward, further research into optimizing these therapeutic strategies will be critical to unlocking the full potential of MAIT cells in clinical applications.

## Conclusion

MAIT cells are pivotal players in bridging innate and adaptive immunity, with their roles extending across health and disease. This review highlights key aspects of MAIT cell biology, including their development, activation mechanisms, functional diversity, and tissue-specific adaptations. While their thymic development and peripheral maturation are defined by distinct markers and transcription factors, their strategic localization in mucosal tissues and barrier sites underscores their importance in immune surveillance and tissue homeostasis. MAIT cells play a crucial role in controlling bacterial and fungal infections through their production of cytokines, including IFN-γ, TNF-α, and IL-17, as well as cytotoxic molecules such as Granzyme B and Perforin. These distinctive features highlight their significance in immune defense and underscore their potential as therapeutic targets for infectious diseases. The activation of MAIT cells, through TCR-dependent and independent pathways, equips them to respond dynamically to microbial metabolites and pro-inflammatory cytokines, emphasizing their dual roles in protective immunity and disease pathogenesis.

Recent advances highlight the potential of MAIT cells in therapeutic immunology. Their unique TCR specificity, abundance in mucosal tissues, and potent effector functions position them as promising candidates for cell-based therapies. Engineering MAIT cells to express CARs targeting tumor or viral antigens could significantly enhance their therapeutic efficacy. The emergence of iPSC-derived MAIT cells offers exciting possibilities for scalable and customizable CAR-MAIT cell therapies. However, challenges such as T cell exhaustion, antigen escape, and the optimization of CAR designs remain to be addressed.

Further research is essential to uncover the intricate tissue-specific roles of MAIT cells, the regulatory interplay with the microbiota, and their potential in immune modulation. By addressing these challenges and expanding our understanding, MAIT cell-based therapies can be refined to unlock their full potential in treating a range of diseases, from infections to cancer and autoimmune disorders, ultimately improving clinical outcomes.

## Data Availability

No datasets were generated or analyzed during the current study.

## Abbreviations

MAIT: Mucosal-associated invariant T cells
TCR: T cell receptor
TRAV: T cell receptor alpha variable region
TRAJ: T cell receptor alpha joining region
TRBV: T cell receptor beta variable region
MHC: Major histocompatibility complex
IBD: Inflammatory bowel disease
MR1: MHC-related protein 1
Vα: α-Chain variable region
Jα: α-Chain joining region
KLF2: Kruppel-like factor 2
S1PR1: Sphingosine-1-phosphate receptor 1
PLZF: Promyelocytic leukemia zinc finger
RORγt: Retinoid-related orphan receptor gamma t
NK: Natural killer cell
IFN-γ: Interferon-γ
MR1-tet: MR1 tetramer
CCR7: C–C chemokine receptor type 7
CCR5: C–C chemokine receptor type 5
CCR6: C–C chemokine receptor type 6
CXCR6: C–X–C chemokine receptor type 6
CCR9: C–C chemokine receptor type 9
TNF-α: Tumor necrosis factor alpha
TLR: Toll-like receptors
CSF2: Colony stimulating factor 2
DN: Double negative
AP-1: Activator protein 1
NF-κB: Nuclear factor kappa-B
EGR1: Early growth response 1
RUNX3: Runt-related transcription factor 3
CCL20: C–C motif chemokine ligand 20
CRC: Colorectal cancer
CAR: Chimeric antigen receptor
VitBAg: Vitamin B-related antigen
CAR-T: Chimeric antigen receptor T-cell
CAR-MAIT: Chimeric antigen receptor mucosal-associated invariant T-cell
CRS: Cytokine release syndrome
EGF: Epidermal growth factor
GM-CSF: Granulocyte–macrophage colony-stimulating factor
iPSC: Induced pluripotent stem cell
5-OP-RU: 5-(2-Oxopropylideneamino)-6-D-ribitylaminouracil
PD-1: Programmed cell death protein 1
CTLA-4: Cytotoxic T-lymphocyte associated protein 4
TIM-3: T-cell immunoglobulin and mucin domain-containing protein 3
HIV: Human immunodeficiency virus
HBV: Hepatitis B
HCV: Hepatitis C
HDV: Hepatitis D
CCL4: C–C chemokine ligand 4
DENV: Dengue virus
ZIKV: Zika virus
SARS-CoV-2: Severe acute respiratory syndrome coronavirus 2
